# Sense of Belonging, Loneliness, and Civic Engagement/ Social Participation Among Japanese and Korean Older Adults

**DOI:** 10.1093/geroni/igaf122.2838

**Published:** 2025-12-31

**Authors:** Li-Mei Chen, Yookyong Lee, Sewon Kim, Sua Jang, Duy Nguyen

**Affiliations:** George Mason University, Fairfax, Virginia, United States; The University of Alabama at Birmingham, Birmingham, Alabama, United States; George Mason University, Fairfax, Virginia, United States; George Mason University, Fairfax, Virginia, United States; Sacred Heart University, Fairfield, Connecticut, United States

## Abstract

Civic engagement/ social participation are not merely activities but are deeply embedded in the psychosocial well-being of older adults. While traditional models of engagement focus on institutionalized activities such as formal volunteering and political participation, they often overlook the emotional, psychological, and relational aspects that drive participation - especially among racial and ethnic minority groups. This study provides a comparative analysis of two Asian ethnic groups—Japanese and Korean older adults in the U.S. examining how cultural and psychosocial factors shape their civic engagement/ social participation and sense of belonging. Using a multi-theoretical qualitative approach and Steps for Coding and Theorization Analysis (SCAT), the in-depth interviews with Japanese (n = 6) and Korean older adults (n = 5) showed that civic engagement/ social participation are tied to their psychological sense of connection to a community. Participation in ethnic associations, informal caregiving, and mutual aid networks fosters a sense of purpose, identity, and emotional security, reinforcing the idea that civic life extends beyond structured activities. On the other hand, language barriers, cultural exclusion, intergenerational conflict, and migration-related social disruptions contribute to loneliness, which in turn diminishes opportunities for engagement and exacerbates emotional distress. Many participants described engagement as a mechanism for reducing isolation and maintaining self-worth, suggesting that civic and social participation serves both as a protective factor and as a coping strategy for aging-related vulnerabilities.

